# Supplementary material for: Identification of genes with oscillatory expression in glioblastoma: the paradigm of SOX2
Source: Sci Rep. 2024 Jan 24;14:2123. doi: 10.1038/s41598-024-51340-z (PMC10808450; doi:10.1038/s41598-024-51340-z)
Supplement: Supplementary file 1 — Supplementary Information. [file 41598_2024_51340_MOESM1_ESM.pdf]

Supplementary Information

Identification of genes with oscillatory expression in glioblastoma: The paradigm of *SOX2*

Fu, R.Z., Cottrell O., Cutillo, L., Rowntree, A., Zador, Z., Wurdak, H., Papalopulu, N*. and Marinopoulou, E.*

* co-corresponding authors

Supplementary Fig. S1

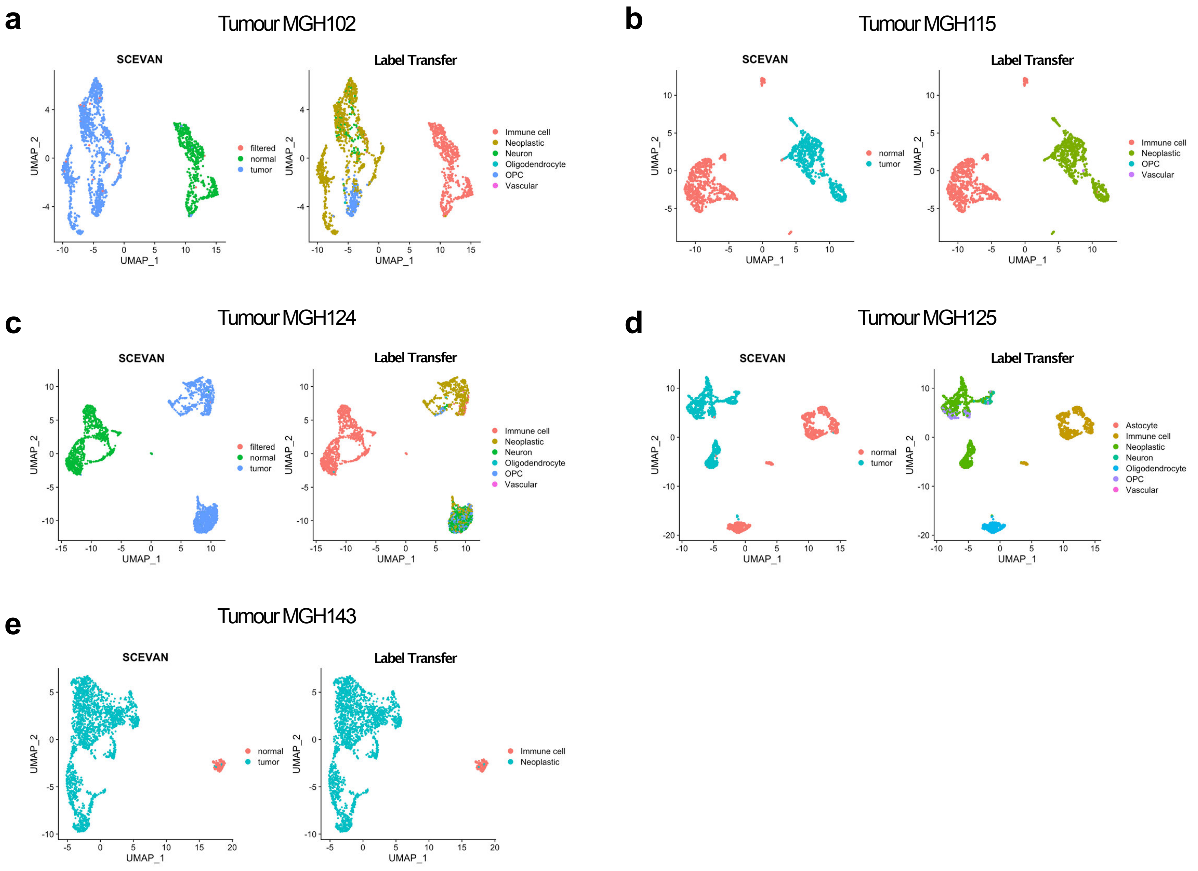

f

Tumour Sample	Total number of cells	Number of "Neoplastic" cells identified by Label Transfer	% of "Neoplastic" cells per tumour	% of "Neoplastic" cells identified as "Tumour" cells by SCEVAN
MGH124	2415	707	29.3	99.9
MGH125	1612	729	45.2	99.6
MGH143	2314	2180	94.2	99.9
MGH102	1822	969	53.2	96.7
MGH115	1283	655	51.1	97.6

Supplementary Fig. S1: Comparison of SCEVAN and Label Transfer methods on GBM tumour data

a-e) UMAP plots representing all single cells in the corresponding tumours and their segregation into different phenotypes based on detection of CNAs using the SCEVAN algorithm and their correspondence to neoplastic and non-neoplastic identities using the Label Transfer method. **f)** Table showing the total number of cells per tumour, the number of cells identified as neoplastic by using the Label Transfer method, the percentage of neoplastic cells within each tumour and the percentage of neoplastic cells also identified as tumour cells by using SCEVAN.

Supplementary Fig. S2

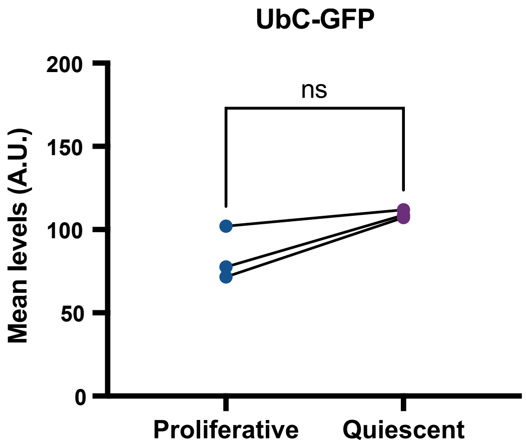

Supplementary Fig. S2: Comparison of UbC-GFP expression levels in proliferative vs quiescent conditions

Graph showing the mean protein expression levels for GFP in proliferative and quiescent conditions, dots represent mean levels per experiment, paired t-test, two-tailed, ns = not significant, n=3 biological replicates, total number of cells tracked: Proliferative = 119, Quiescent = 112

Supplementary Methods

Assessing the effect of zero gene transcription expression on the OscoNet performance

We simulated a synthetic experiment with 100 single cells and 1000 genes, of which 400 were samples from a sinusoidal function with additive Gaussian noise. The 400 oscillators were simulated in 2 frequency groups, each group containing 200 genes. The relative frequencies of the two groups were proportional to 2:3.

In each group, half of the oscillatory genes were simulated as strong oscillators with noise variance σ^2. The other half were simulated as weak oscillators with noise variance (2σ)^2. The starting phase Φ varies in different genes within a frequency group. The remaining genes were simulated as independent Gaussian noise.

To explore the effect of zero gene transcription expression in the overall downstream analysis we varied the percentage of zeros (sparsity) from 0 to 1 with step 0.05, in the expression level of half of the genes in the 100 cells for each of the frequency groups.

For each level of sparsity, we run the OscoNet pipeline and then computed the True Positive Rate (TPR), False Positive Rate (FPR) and False Discovery Rate (FDR) defined as follows and depicted in Supplementary Table S12

TP= number of true co-oscillating pairs
TN= number of true non co-oscillating pairs
FP= number of false co-oscillating pairs
FN= number of false non co-oscillating pairs
FPR= FP/Actual Negative= FP/(FP+TN)
TPR=TP/Actual Positive = TP/(TP+FN)
FDR =FP/ Predicted Positive = FP / (FP + TP)

Increasing the percentage of zeros in the true co-oscillators does not affect the FPR or the FDR that stays close to the suggested threshold 0.05 (Supplementary Fig. S2) Hence, the presence of zeros in our simulated examples does not introduce bias towards the positive discoveries (i.e. number of genes incorrectly identified as co-oscillators). At the same time, we note that an increase of sparsity affects the capability of detecting co-oscillating genes. Indeed, when the sparsity increases from 0 to 0.2, the FPR suffers a fast decrease from 1 to 0.54 while for sparsity greater than 0.2 the FPR falls below 50% (Supplementary Fig. S2). This means that in the pessimistic scenario for which half of the co-oscillating genes in our experiment were affected by at most 20% sparsity, we expect to identify at least 50% of the co-oscillating genes, with an FDR smaller than 0.05. Hence, we suggest 0.2 as sparsity threshold.

	Actual Positive	Actual Negative
Predicted Positive	TP	FP
Predicted Negative	FN	TN

Supplementary Table S13: Confusion matrix representing the intersections between predicted and actual values in terms of TP, TN, FP and FN.

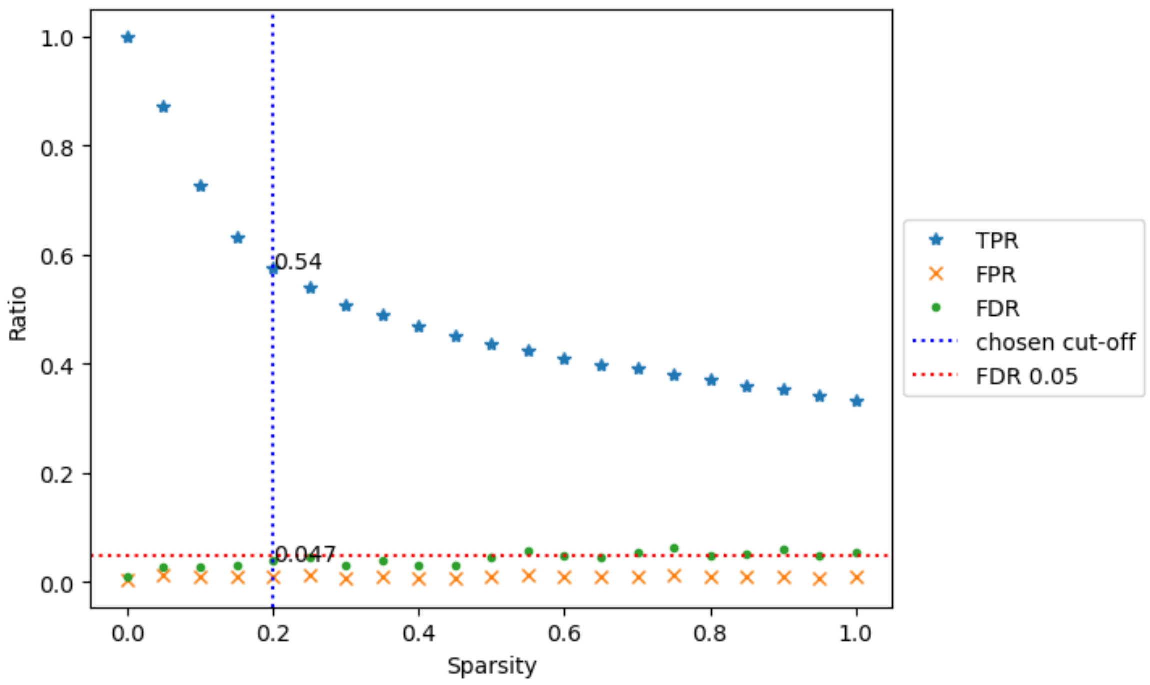

Supplementary Fig. S3: Estimating True Positive Rate, False Positive Rate and False Discovery Rate

Graph showing the TPR, FPR and FDR for every simulated set of data at different level of sparsity [0,…,1]. The vertical dashed blue line represents the suggested cut-off threshold for the maximum number of zeros for a gene to be retained in our analysis. The horizontal red dashed line represents the ideal FRD threshold of 0.05.
